# Prenatal detection of congenital heart disease - results of a Swedish screening program 2013–2017

**DOI:** 10.1186/s12884-021-04028-5

**Published:** 2021-08-22

**Authors:** Maya Waern, Mats Mellander, Anton Berg, Ylva Carlsson

**Affiliations:** 1 Department of Obstetrics and Gynecology, Region Västra Götaland, Sahlgrenska University Hospital, Diagnosvägen 15, Paviljong 7b, 416 85 Gothenburg, Sweden; 2 Pediatric Heart Center, Queen Silvia Children’s Hospital, Sahlgrenska University Hospital, Gothenburg, Sweden; 3 Department of Pediatrics, Institute of Clinical Sciences, Sahlgrenska Academy, University of Gothenburg, Gothenburg, Sweden; 4 Centre of Perinatal Medicine and Health, Institute of Clinical Sciences, Sahlgrenska Aacademy, University of Gothenburg, Gothenburg, Sweden

**Keywords:** Congenital heart defects, Prenatal diagnosis, Screening, Ultrasound, Second-trimester screening

## Abstract

**Background:**

This report evaluates results of a screening program on prenatal detection of congenital heart defects in a geographical cohort of western Sweden between January 1st, 2013 and June 31st, 2017. During the study period 88,230 children were born in VGR.

**Methods:**

Retrospective data on pregnant women from the Västra Götaland region that were referred to fetal cardiologists in Gothenburg were retrieved. To determine prenatal detection rate, all neonates who underwent surgery or catheter intervention for a critical congenital heart defect born between January 1st, 2014 and December 31st, 2016 were included. The four-chamber view was implemented into the routine scan in 2009 and implementation of the ISUOG guidelines, including the outflow tracts, started in the region in 2015.

**Results:**

113 fetuses received a prenatal diagnosis of a major congenital heart defect. 89% of these were referred because of a suspected cardiac malformation and 88% were diagnosed before 22 completed weeks. 59% of the patients diagnosed before 22 completed weeks opted for termination of pregnancy. During 2014–2016, 61 fetuses had a prenatal diagnosis of a critical congenital heart defect and a further 47 were diagnosed after birth, hence 56% were diagnosed prenatally, 82% for those which had a combination with an extracardiac abnormality and/or chromosomal aberration compared to 50% if an isolated critical congenital heart defect was diagnosed. For single ventricle cardiac defects such as hypoplastic left heart syndrome, double inlet left ventricle and tricuspid atresia, the detection rate was 100%. The detection rate for transposition of the great arteries and coarctation of the aorta was 9 and 18% respectively.

**Conclusions:**

56% of all fetuses with a critical congenital heart defect were diagnosed prenatally during 2014–2016 and approximately 53% of all major congenital heart defects 2013–2017 as compared to 13.8% in 2009 in the same region. An increased focus towards the fetal heart in the routine scan improved the prenatal detection rate of major congenital heart defects. The detection of congenital heart defects affecting the four-chamber view seems sufficient, but more training is needed to improve the quality of the examination of the outflow tracts.

**Supplementary Information:**

The online version contains supplementary material available at 10.1186/s12884-021-04028-5.

## Background

Prenatal detection of some types of congenital heart defects (CHD), especially those who demands surgery or catheter intervention within the first 2 months of life (critical congenital heart defects or CCHD) has been shown to have an impact on neonatal survival and morbidity, including neurocognitive outcomes [1–4]. This is most probably a result of avoiding circulatory collapse due to late diagnosis after birth and to planned delivery at specialized centres. Prenatal detection of CHD also allows parents to consider termination of pregnancy (ToP) [5]. In Sweden women are allowed free abortion until the end of the 18th week of the pregnancy and the 22nd week with permission from the National Board of Health and Welfare if special reasons, which could be a cardiac heart defect, are provided. National screening programmes for cardiac defects is part of the screening program in most developed countries and the detection rates vary depending on population screened, staff training and the design of the checklists used [6].

In Sweden 97% of pregnant women participate in the routine ultrasound at 18–20 weeks of gestation, which is offered for free [7]. Region Västra Götaland (VGR) consists of 3 secondary centres and 1 tertiary centre that offer routine scans. These are performed by specially trained midwives and obstetricians. During the period 2013-2017 only 3% of pregnant women attended the routine scan outside these 4 centres (local statistics, not published). In 2009, VGR implemented the four-chamber view of the heart as a mandatory part of routine ultrasounds; in 2013, this procedure became mandatory across Sweden [8]. Following ISUOG guidelines, additional cardiac views were gradually added in VGR to include determination of atrial situs, the right and left outflow tract views and the three-vessel-and-tracheal-view [9]. From 2015 the tertiary centre and one of the secondary centres implemented ISUOG recommendations for fetal heart screening while the other two secondary centres have finished implementation during 2019. Since 2016 this is also mandatory according to national recommendations. The national goals set in 2006 for Sweden aimed at 25% of all major cardiac defects being detected [10], however international data show much higher detection rates [11].

The primary aim of this study was to evaluate the results of the screening program regarding prenatal detection of CHD, with special focus on CCHD, in VGR from January 1st, 2013 until June 31st, 2017. We aimed to analyze detection rates for different types of cardiac defects as well as outcome after a prenatal diagnosis in order to be able to further improve the fetal cardiac screening program in the region. The purpose was to ensure the screening quality against national and international standards and to be able to determine need for further training for the staff as well as to be able to give patients correct information.

## Methods

### Aim, design and setting of the study

This was a retrospective geographical cohort study. Approximately 20,000 infants are born in VGR each year and all fetuses with suspected CHD are referred to the tertiary centre in Gothenburg. Cases were excluded from the study if the mother was not residing in the area during the routine scan.

If the midwife performing the routine scan suspects a cardiac defect, the patient is offered a follow-up examination by a specially trained obstetrician. If there are still deviations from the normal appearance this is considered reason for specialized fetal echocardiography. Pregnancies with an increased risk of CHD are also offered examination by a fetal cardiologist directly (Supplement Table 1).

Patients referred to the fetal cardiologist were examined according to published recommendations [12, 13]. A specially trained sonographer or a fetal cardiologist (paediatric cardiologist specially trained in fetal cardiology) performed the examinations. A fetal cardiologist interpreted all findings. All examinations were performed using ultrasonic equipment with the capacity for 2D, M-mode, colour Doppler and pulsed Doppler. During the study period the examinations were performed on a GE Voluson E8. If the first examination was incomplete and CHD could not be fully excluded, the examination was repeated.

If CHD was diagnosed information was given about the significance, if surgery or other treatment was likely to be necessary after birth, and the expected short and long-term prognosis. The woman/couple then had a visit with an obstetrician, and a plan for the pregnancy was made, including information about the possibility of termination if the diagnosis was made before 22 completed weeks. Amniocentesis to analyse fetal karyotype was offered when indicated. Since 2015 this included analysis for 22q11-deletion.

### The characteristics of participants

We included in the analysis all pregnant women with a fetal diagnosis of CHD between January 1st, 2013 and June 31st, 2017, irrespective of the presence or absence of additional congenital anomalies. Cases of isolated fetal arrhythmias were excluded.

Defects were considered major if they were potentially lethal and/or likely to require surgery or catheter intervention before 12 months of age, and minor when no intervention was likely to be required during the first year. Major heart defects were classified as isolated if there was a normal karyotype and no extracardiac malformations, and otherwise classified as complex.

Within the group of major heart defects, a subgroup of critical congenital heart defects (CCHD) were identified. For the purpose of this study CCHD was defined as CHD requiring surgery and/or catheter intervention within the first 2 months of life in order to avoid serious complications and/or death. In cases of ToP or spontaneous intrauterine fetal death (IUFD) the heart defect was classified as CCHD if the diagnosis usually requires intervention within the first 2 months after birth.

In a subgroup analysis, all neonates with CCHD born between January 1st, 2014 and December 31st, 2016 were included. These were added to the prenatally diagnosed cases with an estimated due date or actual delivery during the same period in order to create a complete cohort of all cases of CCHD during that period. This allowed for calculation of prenatal detection rate of CCHD.

Medical records on all pregnant women in which the fetus was diagnosed with CHD were searched and a number of variables were gathered for each patient (Table 1).

**Table 1:** Prenatally diagnosed major CHD, diagnosis, outcome and chromosomal aberrations /associated malformations

	Total	Diagnosis < 22 w	ToP	IUFD	Postnatal death	Alive at follow-up	Abnormal Karyotype Or Associated Malformations
Total number	113	100	59 (52.2%)	6 (5.3%)	15 (13.2%)	33 (29.2%)	35 (30.9%)
HLHS	35	31	20 (57.1%)	1 (2.9%)	9 (25.7%)	5 (14.3%)	4 (11.4%)
AVSD	23	21	14 (60.8%)	2 (8.7%)	3 (13.0%)	4 (17.3%)	17 (73.9%)
TA	10	10	4 (40.0%)	0	0	6 (60.0%)	1 (10.0%)
ToF	6	6	4 (66.6%)	1 (16.6%)	0	1 (16.6%)	5 (83.3%)
AS	6^a^	4	4 (66.6%)	0	0	2 (33.3%)	0
PA	6	5	4 (66.6%)	0	1 (16.6%)	1 (16.6%)	1(16.6%)
DILV	5	4	2 (40.0%)	0	0	3 (60.0%)	1 (20.0%)
CoA	4^a^	3	1 (25.0%)	0	1 (25.0%)	2 (50.0%)	1 (25.0%)
Truncus arteriosus	3	2	1 (33.3%)	0	1 (33.3%)	1 (33.3%)	2 (66.7%)
DORV	3	2	1 (33.3%)	0	0	2 (66.7%)	1 (33.3%)
dTGA	3	3	0	0	0	3 (100%)	0
ccTGA	2	2	0	0	0	2 (100%)	0
Ebstein’s anomaly	2	2	0	2 (100%)	0	0	0
TS	2	2	2 (100%)	0	0	0	1 (50.0%)
Left ventricular hypoplasia	2	2	2 (100%)	0	0	0	1 (50.0%)
PS	1	1	0	0	0	1 (100%)	0

ToP, Termination of Pregnancy; IUFD, Intrauterine fetal death; CHD, Congenital heart disease; HLHS, Hypoplastic left heart syndrome; AVSD, Atrioventricular septal defect; TA, Tricuspid atresia; ToF, Tetralogy of Fallot; AS, Aortic stenosis; PA, Pulmonary atresia; DILV, Double inlet left ventricle; CoA, Coarctation of the aorta; DORV, Double outlet right ventricle; dTGA, dextro Transposition of the great arteries; ccTGA, congenitally corrected Transposition of the great arteries; TS: Tricuspid stenosis; PS, Pulmonary stenosis

^a^Two of the patients with AS were prenatally diagnosed as CoA. These have been sorted as AS in the Table

### Statistical analyses

This study was largely descriptive rather than comparative; hence median and range were used for continuous variables and percentages for categorical variables. Groups were compared using Fishers exact test. The level of significance was set at *p* < 0.05. The median follow-up of live births was 2 years, with a range of 3 weeks to 4 years.

## Results

### General characteristics

During the study period 88,230 children were born in VGR. Of these, 1274 women (1303 fetuses) were referred to and examined by a fetal cardiologist and of these, 1073 examinations (84%) were performed before 22 completed weeks (median 19.4, range 14–41 weeks).

The most common reason for referral was family history, accounting for 48% of all examinations (Supplement Table 1), but only 0.3% of these fetuses were found to have CHD. In the group referred because of a suspected cardiac malformation, 41% were found to have CHD.

Prenatally 130 fetuses were diagnosed with CHD, 110 of whom were examined before 22 completed weeks (85%). 113 out of 130 had major CHD, six had minor CHD and seven could not be classified during pregnancy. In four cases the diagnosis of a major CHD could not be confirmed on fetal autopsy or postnatally.

Of the 113 patients found to have major CHD, 102 (90%) were referred because of a suspected cardiac malformation, others due to extra-cardiac abnormalities (five), family history (three, in all cases on the mother’s side), increased nuchal translucency > 3.5 mm (NT) (three) and maternal risk factors (one, diabetes with poor metabolic control). There were 78 fetuses with a single ventricle heart, and of these 72 were diagnosed before 22 completed weeks (92%).

5.2% were referred because of increased nuchal translucency (> 3.5 mm), and 4.1% of these were diagnosed with CHD.

Three additional patients with suspected CHD were found at the routine scan, all at the tertiary centre, but, due to multiple malformations and chromosomal aberrations (two trisomy 13, one trisomy 21), the pregnancies were terminated without referral to the fetal cardiologist. These are not further included in the report, since no definitive diagnosis of CHD was made.

### Unclassified CHDs

Seven fetuses diagnosed with CHD could not be classified as major or minor during pregnancy. In this group there was one with suspected cardiomyopathy in a duplex-pregnancy, both twins normal at birth. One case had ectopia cordis, and the parents chose ToP before any cardiac defect could be confirmed. In one case aplasia of ductus venosus was diagnosed together with an abnormal course of the umbilical vein to the superior vena cava and MRI showed that the portal vein was missing. The pregnancy was terminated and findings confirmed at autopsy. One fetus with a suspicion of VSD suffered IUFD, the fetal karyotype was 69XXX and autopsy revealed multiple malformations, but could not confirm the VSD. One fetus with suspected primum ASD was postnatally found to have total anomalous pulmonary venous return to the coronary sinus. This fetus also had “cat eye-syndrome”, and died shortly after birth. In one patient there was a suspicion of ductal constriction at 37 completed weeks, however examination postnatally was normal. In one patient referred because of diaphragmatic hernia the fetal cardiologist suspected a possible univentricular heart malformation, but due to the hernia this could not be classified. The child died soon after birth and autopsy showed multiple malformations and hypoplasia of the right ventricle.

### Minor CHDs

Six fetuses were diagnosed with minor CHD. One fetus with right aortic arch and vascular ring and one with cardiomyopathy were confirmed postnatally, while two had a structurally normal heart (suspicion of cardiomyopathy as well as a pulmonary valve stenosis). One with prenatal suspicion of cardiomyopathy had a patent ductus arteriosus which required surgery, but no cardiomyopathy could be confirmed. In one case with prenatally diagnosed VSD, autopsy after IUFD showed an enlarged heart but no VSD. These six cases were not further analysed.

### Major CHDs

**Fig. 1:**
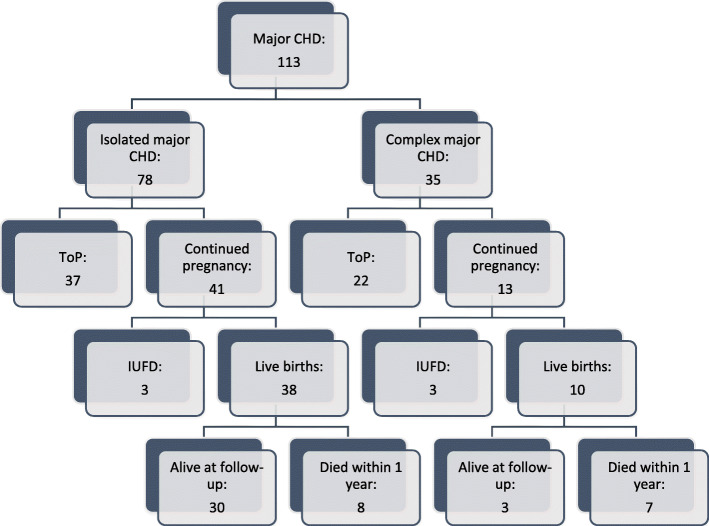
Pregnancy outcome in foetuses diagnosed with isolated or complex major congenital heart disease; CHD (with or without a chromosomal aberration and/or extracardiac malformation). (CHD, Congenital heart disease; ToP, Termination of Pregnancy; IUFD, Intrauterine fetal death)

Major CHD was diagnosed in 113 fetuses (Table 1). Of 100 that were diagnosed before 22 completed weeks 59 (59%) opted for ToP (Fig. 1). In 54 continuing pregnancies, six fetuses died in utero and 48 were born alive. Fifteen died within the first year and 32 were still alive at follow-up. The total mortality, ToP excluded, was 38,9% (21 of 54). Of those diagnosed before 22 weeks, 90/100 had an amniocentesis. The number of patients with an associated malformation or chromosomal aberration was 35/113 (31%) for major CHD, 17/78 (22%) for single ventricle lesions and 18/35 (51%) for major biventricular cardiac defects (Table 1).

Intrauterine transport to the tertiary centre was considered in those cases where the cardiac defect was predicted to be potentially life-threatening to the neonate, the most common reason being duct dependent pulmonary or systemic circulation. Out of 54 continuing pregnancies with fetal major CHD, 21 were from a secondary centre. One fetus died before a decision was made whether or not to relocate the delivery. One woman had premature rupture of membranes at 24 completed weeks and was not transported to the tertiary centre due to the grave prognosis. Three patients were not recommended delivery at the tertiary centre, in one case because of poor prognosis. In the other two cases further examinations were planned, but one delivered prematurely at 33 weeks and one was transported after start of premature labor and delivered at the tertiary centre at 28 weeks. The remaining 16/21 women were recommended to deliver in the tertiary centre. One of the 16 fetuses died in utero, the other 15 were born in the tertiary centre. Of these, 13 were diagnosed with a duct-dependent CHD.

### False positives

Fetal autopsy was performed in 30 of the 59 fetuses that were terminated. In 28 cases autopsy confirmed the prenatal diagnosis. In one case the prenatal diagnosis of AVSD was uncertain. A second examination was planned, but the pregnancy was terminated because of extracardiac malformations. The autopsy confirmed multiple extracardiac malformations, but could not confirm the AVSD. One patient prenatally diagnosed with fetal HLHS opted for termination of pregnancy, and fetal autopsy could not confirm the diagnosis. The images were reviewed by two fetal cardiologists, but were of poor quality and inconclusive. This patient is not included in Fig. 1, Tables 1 or 2. Two fetuses, who were diagnosed prenatally with CoA were normal on postnatal examination. These are not included in Fig. 1, Tables 1 or 2. This is equivalent to a false positive rate of 0.2%.

**Table 2:** Pre- and postnatally detected cases of CCHD 2014–2016

Group	Total number	Prenatal	Prenatal (%)	ToP	ToP % of prenatal before 22 weeks	IUFD	Liveborn	Alive at follow-up
CoA	17	3	18%	1	33%	0	16	15
TGA	11	1	9%	0	0%	0	11	11
HLHS	23	23	100%	13	57%	1	9	4
DILV	5	5	100%	2	40%	0	3	3
TA	5	5	100%	3	60%	0	2	2
Other	47	24	51%	17	71%	2	28	24
Total	108	61	56%	36	59%	3	69	59

*CCHD* Critical congenital heart defects, *ToP* Termination of Pregnancy, *IUFD* Intrauterine fetal death, *CoA* Coarctation of the aorta, *TGA* Transposition of the great arteries, *HLHS* Hypoplastic left heart syndrome, *DILV* Double inlet left ventricle, *TA* Tricuspid atresia

### Subgroup analysis of all prenatally and postnatally diagnosed cases of CCHD 2014–2016

Sixty-one fetuses were considered to have CCHD (defined as described in Methods) and an estimated due date or actual delivery date during 2014–2016. These 61 cases were analysed together with all children postnatally diagnosed with CCHD during 2014 to 2016. In total 108 fetuses/children with CCHD were identified, and thus 61 had a prenatal diagnosis (56%). The prenatal detection rate was 18/22 (82%) for those who had CCHD in combination with an extracardiac abnormality and/or chromosomal aberration compared to 43/86 (50%) in those with isolated CCHD (Table 2).

Of the 61 cases with a prenatal diagnosis, 36 opted for TOP and three fetuses died in utero. Hence 22 were born alive and of these 13 were still alive at follow-up. In this group 18/61 (29%) had an associated chromosomal aberration or extra cardiac malformation, and of those born alive the corresponding number was 5/22 (23%). Two of the 13 still alive at follow-up have an associated chromosomal aberration or malformation.

There were 47 cases of CCHD diagnosed postnatally, all but one of these were still alive at follow-up. Four have an associated chromosomal aberration or associated malformation (8.5%).

In the prenatally detected group, 13/61 were alive at follow-up. If we exclude ToP, 13/25 prenatally diagnosed with CCHD were alive at follow-up, compared to 46/47 in the group postnatally detected (*P* < 0.0001).

## Discussion

The number of pregnant women being referred for specialized fetal echocardiography at Sahlgrenska University Hospital because of a suspicion of CHD in the fetus increases year by year. As a result, the number of cases of CHD detected prenatally gradually increases. In total, 24% were referred because of suspected CHD and 87% of all cases diagnosed with CHD were in this group, corresponding well with previously published data [13]. Approximately half of all women examined by the fetal cardiologist were referred because of family history, but only 2,6% of all major cardiac defects detected were found in this group, in all cases the mother was affected [13].

The definition of CCHD varies in the literature. Some have used a one-month limit or focused only on those requiring an emergency procedure within the first few hours of life (mainly TGA or HLHS with severely restrictive atrial communication and TAPVR with obstructed pulmonary venous return) [14, 15]. We used a 2 month limit in order to include most cases of critical CoA, since this is the critical cardiac defect most commonly missed by postnatal screening using pulse oximetry [16]. There was 100% prenatal detection for some CCHDs, all of which were defects affecting the four-chamber view, such as HLHS, DILV and TA. The detection rate for other CCHDs was low, especially for those not associated with an abnormal four-chamber view, such as TGA (9%), or CHDs with a progressive evolution that are not always detectable during the second trimester such as CoA (16%). Extended heart screening was only practiced during the latter part of the study and is still not fully introduced in two of the three secondary centres. A more extensive screening program and staff training has been shown to improve TGA detection rate extensively from 14 to 77% [17]. The detection of TGA was better during the subsequent part of the study period; only 2 TGA were detected during the first 3 years of the study and 4 during the last year. This likely reflects the continuing training of midwives performing the routine scans in VGR as well as a more extensive checklist especially concerning the heart in the latter years. CoA is known to be difficult to diagnose prenatally [16, 18], with both missed cases and false positives being a problem. It is troublesome that so few fetuses with TGA and CoA are diagnosed prenatally, since these diagnoses are among those in which a prenatal diagnosis is most beneficial [1, 2, 4]. The absence of a centralized steering of the screening program in Sweden and hence the possibility of centres not to fully adhere to a national guideline is problematic and something that needs to be addressed on a national level along with the implementation of a national quality follow-up program.

The detection rates achieved in other reports are also widely divergent, varying from 5 to 92% [19], indicating the difficulties in screening for CHDs. A number of factors affect the detection rate at the routine scan, such as the number of ultrasound scans during pregnancy, gestational age at the time of the routine scan, the position of the fetus, the BMI of the mother, the experience of the ultrasound operator, the time allocated for the scan and which checklist is used [19].

The two main purposes of a screening program for cardiac defects in the fetus is to increase the chances of intact postnatal survival and to ensure the autonomy of the pregnant woman [20]. A high-quality screening program will detect most cases of CCHD and avoid false positive diagnoses. Although the detection rate of cardiac defects gradually increases, there are clear regional differences both within a country as well as internationally and hence results in an ethical dilemma which needs to be addressed by continuous education and training. The overall false positive rate in this study was 0.2%, well in line with other reports [21], however there was one case where the patient opted for ToP and the diagnosis could not be confirmed on post-mortem examination. While an inexperienced examiner or obesity could have caused this false positive result, it is something that should be avoided by all means by continuous education and training of staff involved in the screening.

According to Statistics Sweden, 58,930 babies were born in VGR during 2014–2016. During this period 108 cases with CCHD were identified, including 3 IUFDs and 37 ToP. This translates to a fetal incidence of 1.8/1000, which is consistent with the expected incidence [22] and supports the notion that most cases were identified. The incidence at live birth was 1.2/1000. Cases not diagnosed prenatally and suffering IUFD, where the pathological examination might have revealed a heart defect were not included and we did not search cases born alive without a prenatal diagnosis, but not surviving to have surgery or catheter intervention. Of the identified fetuses and children with CCHD 56% had a prenatal diagnosis. In a recently published study from the US a prenatal detection rate of 78% was reached using serial scans, which enabled birth at the congenital cardiac unit in 92% of the cases and hence decreasing neonatal transports [23]. However, in Sweden a majority of pregnant women only go through one ultrasound during their pregnancy increasing the importance of a good quality routine scan.

Extrapolating from an estimated fetal prevalence of 2.4/1000 having major CHD [24], 212/88230 fetuses should have had major CHD during our study period. During this period 113 cases of major CHDs were identified prenatally, 56 of these were terminated and 6 fetuses died in utero. The prenatal detection rate of major CHDs could thus be estimated at approximately 53%. Compared to the results in the same region of Sweden for the period 1989–2002 [25], during which approximately 11.4% of all major CHDs were detected, or the results for the period 2002–2009 [26] where approximately 13,8% of all major CHDs were identified, there has been a significant improvement in detection rates since the start of CHD screening in 2009. Twelve of 53 cases of HLHS were diagnosed prenatally between 2002 and 2009, as compared to 100% between 2013 and 2017. The increased detection of major CHDs depends largely on the training of midwives performing the routine scan and a more extensive checklist [27].

The overall termination rate was 59% in the group with major CHDs diagnosed before 22 completed weeks and 64% when a univentricular cardiac defect was diagnosed. The corresponding number for univentricular cardiac defects from the Stockholm area during 2007–2014 was 70% [28] and 52% for major CHDs in Finland [6]. Terminations because of CCHD constituted 33% of the total population of fetuses and neonates with CCHD, lower than in some other parts of the nation [21] indicating a lower prenatal detection rate. In the group that were prenatally diagnosed with CHD considered to be suitable for biventricular repair, and without an abnormal karyotype or associated malformation, zero pregnancies were terminated.

All patients opting for termination were offered fetal autopsy, however only 50% accepted. Improving this proportion would be valuable in order to ensure the quality of screening.

In the group of CCHDs diagnosed prenatally, 31% had a chromosomal aberration or associated malformation and 84% were univentricular defects. The corresponding proportions for children diagnosed postnatally was 8.1 and 6.4% respectively. Similar results have been reported by others [29, 30]. Outcome was significantly better in the group diagnosed postnatally. This difference is largely explained by the fact that the prenatally diagnosed cases consist to a large extent of single ventricle lesions and have a high incidence of associated malformations and chromosomal aberrations. The postnatally diagnosed children have lesions such as TGA and CoA with an excellent prognosis.

It is well known that an abnormal karyotype and extra cardiac malformations are strongly associated with CHDs [13, 31]. In our study, 30% of major CHDs had an associated extracardiac malformation or chromosomal aberration, which corresponds well with other reports [29, 32].

### Strength and limitations

One major strength of the study is the large population of pregnant women from a well-defined geographical area, of whom 97% undergo the second trimester routine scan. Having only one tertiary centre and one fetal cardiology unit enabled us to follow up on all patients who were examined by fetal cardiologists during the study period.

This report misses the cases that were not detected prenatally and died during pregnancy or after birth before surgery. Wren et al. (2008) found that about 5% of children affected by CCHD died undiagnosed [33]. However, since 2014 pulse oximetry screening is used on all new-borns in Sweden thereby minimizing the number of missed cases. This indicates that missed cases who died before surgery could not be expected to significantly alter the numbers in this study.

Another limitation is that for the subgroup analysis including postnatally diagnosed cases only CCHD were included, while the number of major CHDs born during that period was not searched. Therefore, the more precise prenatal detection rate could only be analysed for CCHDs, while the prenatal detection rate for CHDs is estimated.

## Conclusions

Our data indicates that the training of midwives performing the routine scan and the incorporation of a more extensive heart scan as part of the routine scan has had an effect on the prenatal detection rate of major CHDs. However, as of 2019, the full scan recommended by ISUOG [9] had not been fully implemented in the whole region, which is reflected by the numbers of prenatally detected cases in this study. The numbers seem to indicate that the detection of CHDs based on the four-chamber view is sufficient, but that education concerning the outflow views and the three-vessel-and-tracheal-view in all screening units in VGR is of outmost importance.

## Supplementary Information

**Additional file 1.**

## Data Availability

The datasets generated and/or analysed during the current study are not publicly available due confidentiality under Swedish law, but are available encoded from the corresponding author on reasonable request.

## Abbreviations

CHD: Congenital heart disease
ToP: Termination of Pregnancy
VGR: Region Västra Götaland
ISUOG: International Society for Ultrasound in Obstetrics and Gynecology
CCHD: Critical congenital heart defects
IUFD: Intrauterine fetal death
NT: nuchal translucency
MRI: Magnetic resonance imaging
VSD: Ventricular septal defect
AVSD: Atrioventricular septal defect
CoA: Coarctation of the aorta
dTGA: dextro Transposition of the great arteries
cTGA: corrected Transposition of the great arteries
HLHS: Hypoplastic left heart syndrome
DILV: Double inlet left ventricle
TA: Tricuspid atresia
ASD: Atrial septal defect
TAPVR: Total anomalous pulmonary venous return
ToF: Tetralogy of Fallot
AS: Aortic stenosis
PA: Pulmonary atresia
DORV: double outlet right ventricle
TS: Tricuspid stenosis
PS: Pulmonary stenosis
HAA: Hypoplastic aortic arch
