# Supplementary material for: Prenatal detection of congenital heart disease - results of a Swedish screening program 2013–2017
Source: BMC Pregnancy Childbirth. 2021 Aug 22;21:579. doi: 10.1186/s12884-021-04028-5 (PMC8380393; doi:10.1186/s12884-021-04028-5)
Supplement: Supplementary file 1 — Additional file 1. [file 12884_2021_4028_MOESM1_ESM.docx]

**Supplement Table 1.** Indications for examination by a fetal cardiologist.

| Indication | First visit, total number | CHD diagnosed |
| --- | --- | --- |
| Suspected CHD | 281 (22.1%) | 113 |
| Family history | 612 (48.0%) | 3 |
| Fetal arrhythmia | 75 (5.9%) | 0 |
| Extracardiac abnormalities | 69 (5.4%) | 7 |
| Abnormal karyotypes | 1 (0.1%) | 0 |
| Hydrops fetalis | 11 (0.9%) | 1 |
| Increased NT | 72 (5.7%) | 3 |
| Maternal risk factor | 131 (10.3%) | 1 |
| Other | 22 (1.7%) | 2 |
| Total | 1274 | 130 |

CHD, Congenital heart disease; NT, nuchal translucency;
